# Safety and efficacy of robot-assisted percutaneous kyphoplasty under local anesthesia in a day-surgery setting for osteoporotic vertebral compression fractures

**DOI:** 10.3389/fsurg.2025.1595231

**Published:** 2025-07-28

**Authors:** Yaming Wu, Yang Yuan, Yi Yin, Yaqin Gong, Yaowei Ye, Li Wu, Lizhen Qian

**Affiliations:** ^1^ Department of Spine Surgery, The Affiliated Kunshan Hospital of Jiangsu University (The First People's Hospital of Kunshan), Kunshan, Jiangsu, China; ^2^ Department of Urology, Panzhihua Central Hospital, Panzhihua, Sichuan, China; ^3^ Office of Science and Technology, The Affiliated Kunshan Hospital of Jiangsu University (The First People’s Hospital of Kunshan), Kunshan, Jiangsu, China; ^4^ Department of Anesthesiology and Operating, The Affiliated Kunshan Hospital of Jiangsu University (The First People’s Hospital of Kunshan), Kunshan, Jiangsu, China

**Keywords:** osteoporotic vertebral compression fractures, day surgery, robotics, percutaneous kyphoplasty, local anesthesia

## Abstract

**Background and objectives:**

Osteoporotic vertebral compression fractures (OVCFs) represent a growing healthcare challenge in aging populations. This retrospective study evaluates the safety and efficacy of robot-assisted percutaneous kyphoplasty (PKP) performed under local anesthesia within a day-surgery framework.

**Methods:**

Clinical data from 127 patients with OVCFs who underwent robot-assisted PKP under local anesthesia in a day-surgery setting at the First People's Hospital of Kunshan between May 2022 and April 2024 were retrospectively analyzed. The cohort comprised 40 males and 87 females, with a mean age of 67.1 ± 6.8 years and a mean body mass index (BMI) of 22.5 ± 3.0 kg/m². The prevalence of comorbidities was as follows: diabetes (*n* = 36), hypertension (*n* = 55), coronary heart disease (CHD) (*n* = 28), and chronic obstructive pulmonary disease (COPD) (*n* = 15). Data collection included the following parameters: pain intensity (Visual Analog Scale, VAS), Oswestry Disability Index (ODI), operative time, cement volume, complications, length of hospital stay, and hospitalization costs.

**Results:**

All procedures were successfully completed under local anesthesia with a mean operative time of 56.1 ± 10.2 minutes. The robotic system demonstrated high precision (first-attempt puncture success rate: 95.3%). Significant clinical improvements were observed: VAS scores decreased progressively from 7.9 ± 1.1 to 1.3 ± 0.3 at 6 months [*F*(4,504) = 386.2, *p* < 0.001, *η*² = 0.75], representing an 83.5% improvement, while ODI scores improved by 77.1% [75.2% → 17.2%, *F*(4,504) = 412.8, *p* < 0.001, *η*² = 0.77]. Cobb angle correction reached 5.4° [95%CI:4.1–6.7°, *t*(126) = 12.6, *p* < 0.001, *d* = 1.12]. The complication rate was 4.8% (cement leakage: 2.4%; transient hypotension: 0.8%). Patients were discharged within 34 ± 4.3 h postoperatively, with mean hospitalization costs of 37,100 ± 4,200 RMB. No 30-day readmissions occurred.

**Conclusion:**

Robot-assisted PKP under local anesthesia in day surgery demonstrates excellent safety and efficacy for OVCF management. This approach combines robotic precision with accelerated recovery pathways, particularly benefiting elderly patients with comorbidities.

## 1 Introduction

OVCFs represent a prevalent type of fragility fracture, constituting a significant global health concern (1). The annual incidence of OVCFs is rising due to the aging global population, with estimates of 120,000 cases per year in the UK and approximately 750,000 new cases annually in the United States. Epidemiological studies indicate an annual incidence of 307 per 100,000 individuals over 50 years of age, with a notably higher occurrence in women (50%) compared to men (20%). Specifically, women aged 85–89 exhibit an incidence approximately eight times greater than that of women aged 60–64 (2–5). Beyond causing severe pain, OVCFs significantly impair patients’ mobility and quality of life and, in severe cases, can lead to multiple organ failure and mortality (6). Furthermore, OVCFs impose a substantial socioeconomic burden, with direct costs for a first-time fracture estimated at approximately €6490 (7). The cost of managing fragility fractures in the UK was approximately £2.3 billion in 2011 and is projected to exceed £6 billion by 2036 (2, 6). The elevated incidence, potential for complications, and considerable economic impact of OVCFs underscore their importance as a critical public health challenge.

Traditional treatment approaches for OVCFs encompass conservative management (e.g., bed rest, bracing, and analgesic medications) and open surgery. Conservative management often exhibits limited effectiveness, with prolonged bed rest potentially leading to complications such as pneumonia, pressure ulcers, and deep vein thrombosis. Open surgery carries significant trauma and risks, rendering it unsuitable for many elderly patients with comorbidities (8, 9). PKP, a minimally invasive alternative, offers several advantages, including minimal trauma, rapid recovery, shorter operative times, reduced blood loss, and prompt clinical efficacy, establishing it as a preferred method for OVCF management (8, 10–12). The advent of the day-surgery model, driven by advances in medical technology, presents an alternative approach. While OVCFs are not invariably emergency cases, timely and effective intervention can substantially enhance the quality of life for patients experiencing severe pain. Consequently, the application of an “emergency day surgery” framework can be considered, facilitating a safe and efficient surgical pathway wherein patients are discharged within 24–48 h of admission (13). Robot-assisted technology can augment the precision and safety of PKP, mitigating the incidence of complications such as cement leakage (12, 14). Concurrently, performing the procedure under local anesthesia circumvents the risks associated with general anesthesia, particularly benefiting elderly and comorbid patients by decreasing surgical risk and expanding treatment accessibility (14, 15). Integrating the day-surgery model with robot-assisted technology and local anesthesia optimizes the reduction of hospital stays, diminishes medical costs, enhances medical efficiency, and facilitates patients’ earlier return to their families and communities, aligning with contemporary healthcare initiatives. The present study retrospectively analyzed clinical data from OVCF patients undergoing robot-assisted PKP under local anesthesia within a day-surgery setting at the First People's Hospital of Kunshan to evaluate the safety and effectiveness of this integrated approach and highlight the benefits of local anesthesia and the day-surgery model.

## 2 Methods

### 2.1 Inclusion and exclusion criteria

**Inclusion Criteria:**

(1) Age ≥55 years;
(2) Fresh osteoporotic vertebral compression fractures in the T5-L5 segment, confirmed by imaging, with no neurological deficits;
(3) Number of fractures ≤3;
(4) American Society of Anesthesiologists (ASA) physical status classification I-III;
(5) Signed informed consent, agreeing to undergo day-surgery treatment.

**Exclusion Criteria:**

(1) Pathological fractures (e.g., fractures caused by tumors);
(2) Severe cardiopulmonary insufficiency, unable to tolerate surgery;
(3) Coagulation disorders;
(4) Local infection at the puncture site;
(5) Mental illness or cognitive impairment, unable to cooperate with treatment;
(6) Allergy to local anesthetic drugs.

### 2.2 General data

A total of 127 patients were included in this study. All patients were diagnosed with osteoporosis (T-score ≤−2.5) by dual-energy x-ray absorptiometry. Preoperative evaluations included electrocardiogram, chest CT, spinal MRI, complete blood count, coagulation function tests, liver and kidney function tests, blood glucose, and blood pressure monitoring. All patients and their families understood the day-surgery procedures and signed informed consent forms. The study protocol received ethical approval from the Institutional Review Board of The First People's Hospital of Kunshan City (2020-03-046-K01).

### 2.3 Surgical technique

i. Positioning and Surgical Site Preparation: With the patient in a prone position, a square silicone pad was used to elevate the chest and pelvis, allowing the abdomen to hang freely, facilitating manual fracture reduction. Reduction was then verified fluoroscopically using a C-arm. The surgical site was prepared routinely with an iodine-based antiseptic solution, draped, and covered with a sterile adhesive film (3M).
ii. Robot-Assisted Positioning: An external optical tracker was affixed to the skin distally to the surgical site using sterile adhesive film (3M), ensuring unobstructed visibility of the optical tracking area. The robotic arm optical tracker (Tiantu robot) was then positioned. Three-dimensional volumetric scanning was performed via C-arm fluoroscopy, and this data was uploaded to the robotic system for three-dimensional model generation. Based on preoperative imaging and fracture characteristics, unilateral transpedicular puncture points, trajectories, and target locations within the vertebral body were planned.
iii. Local Anesthesia and Puncture: The skin entry point was determined with robotic guidance. Local infiltration with 1% lidocaine was performed, followed by transverse process nerve blocks. A 5 mm skin incision was created. The entry point was re-verified under robotic guidance to confirm the absence of displacement. A Kirschner wire was then advanced through the guide to the bone surface and gently tapped approximately 3 mm into the bone, ensuring minimal resistance. Anteroposterior and lateral C-arm fluoroscopy confirmed accurate Kirschner wire placement.
iv. Channel Creation and Vertebral Reduction: The robotic system was detached. A working channel was created over the Kirschner wire, which was subsequently removed. A routine bone biopsy was obtained, and the tissue submitted for pathological analysis. One milliliter of local anesthetic was injected into the vertebral body, followed by gentle tapping. A balloon dilator was inserted to expand the collapsed endplate, reducing the vertebral body and, simultaneously, compressing the fracture line to minimize cement leakage. The degree of reduction was assessed fluoroscopically using a C-arm.
v. Bone Cement Injection: Bone cement was mixed to a stringy consistency. Using a pusher, bone cement was slowly injected from distal to proximal under real-time fluoroscopic monitoring to assess cement dispersion. Injection was ceased upon achieving optimal fill.
vi. Postoperative Management: Following cement solidification, the puncture cannula was removed. Local pressure was applied to achieve hemostasis, and a sterile dressing was applied to the incision site.

### 2.4 Perioperative management

(1) All patients received standardized perioperative management, including detailed preoperative education on the procedure, anesthesia, precautions, pain management, and rehabilitation. Day-surgery characteristics, discharge criteria, and tolerance of local anesthesia were assessed.
(2) The Kunshan Fracture Liaison Service (FLS) assessed patients for osteoporosis risks and developed individualized management plans, including lifestyle guidance, supplementation, and medication recommendations to reduce refracture risk.
(3) Postoperatively, vital signs, pain (VAS), neurological function, and the puncture site were closely monitored. Patients were encouraged to ambulate with a brace 4–6 h postoperatively and received supervised rehabilitation. Spinal x-rays were performed 6–8 h postoperatively to assess discharge readiness. Postoperative precautions were reinforced before discharge. Patients were followed up via telephone at 1 day, 1 month, 3 months, and 6 months to monitor recovery, pain control, complications, and osteoporosis management, with follow-up visits scheduled as needed.

### 2.5 Observation indicators

i. **Baseline Patient Characteristics:** Gender, age, body mass index (BMI), comorbidities (diabetes, hypertension, CHD, COPD, etc.), bone mineral density (BMD T-score), fracture location and number, American Society of Anesthesiologists physical status classification.
ii. **Surgical Parameters:** Operative time, bone cement injection volume, fluoroscopy times.
iii. **Clinical Efficacy:** Pain and function were assessed using the VAS and ODI preoperatively and at 1 day, 1 month, 3 months, and 6 months postoperatively. Cobb angle was recorded preoperatively and at 1 day and 6 months postoperatively.
iv. **Complications:** Including bone cement leakage, infection, hemorrhage, hematoma, nerve injury, local anesthesia complications, etc., as well as readmission within 30 days postoperatively.
v. **Hospitalization and Costs:** Length of hospital stay and total hospitalization costs were recorded.

### 2.6 Statistical analysis

All statistical analyses were performed using SPSS 27.0 (IBM Corp). Continuous variables were assessed for normality using the Shapiro–Wilk test. For parameters measured at multiple time points (VAS, ODI):

1. Repeated Measures ANOVA was employed to evaluate overall time effects, with *post hoc* pairwise comparisons using Bonferroni correction. Sphericity assumption was verified by Mauchly's test (*α* = 0.05); Greenhouse-Geisser adjustment applied when violated.
2. Paired t-tests analyzed Cobb angle changes between preoperative, postoperative day 1, and 6-month measurements (due to missing 1-/3-month data).

Effect sizes were reported as partial *η*² (>0.14 medium, >0.40 large) for ANOVA and Cohen's d (>0.50 medium, > 0.80 large) for t-tests. Data presented as mean ± SD unless specified. Missing Cobb angle values (postoperative 1/3 months) were excluded without imputation. Statistical significance was set at two-tailed *p* < 0.05 with multiplicity-adjusted thresholds.

## 3 Results

### 3.1 Patient demographics and baseline characteristics

Table 1 summarizes the demographic and baseline characteristics of the 127 patients. The cohort included 40 males and 87 females, with a mean age of 67.1 ± 6.8 years and a mean BMI of 22.5 ± 3.0 kg/m². Comorbidities included diabetes (28.3%), hypertension (43.3%), CHD (22.0%), and COPD (11.8%). The mean BMD T-score was −3.1 ± 0.4. Fracture locations were thoracic (45), lumbar (48), and thoracolumbar (34). The number of fractures was one (98), two (21), and three (8).

**Table 1:** Baseline characteristics of patients.

Characteristic	Number of patients (n)	Gender (Male/Female)	Age (years)	BMI (kg/m²)	Diabetes (*n*)	Hypertension (*n*)	CHD (*n*)	COPD (*n*)	BMD T-score	Fracture location (Thoracic/Lumbar/Thoracolumbar)	Fracture number (1/2/3)
Value	127	40/87	67.1 ± 6.8	22.5 ± 3.0	36	55	28	15	−3.1 ± 0.4	45/48/34	98/21/8

### 3.2 Surgical parameters

Surgical parameters are presented in Table 2. The mean preoperative preparation time was 16.5 ± 4.1 min, operative time was 56.1 ± 10.2 min, and channel establishment time was 11.0 ± 3.0 min. The mean bone cement injection volume was 6.5 ± 1.5 ml, the mean fluoroscopy dose was 37.1 ± 8.8 mGy, and the mean fluoroscopy count was 6.8 ± 1.7. The initial puncture success rate was 95.3%.

**Table 2:** Surgical parameters.

Surgical Parameter	Preoperative Preparation Time (minutes)	Operative Time (minutes)	Channel Establishment Time (minutes)	Bone Cement Injection Volume (ml)	Fluoroscopy Dose (mGy)	Fluoroscopy Count	Initial Puncture Success Rate (%)
Value	16.5 ± 4.1	56.1 ± 10.2	11.0 ± 3.0	6.5 ± 1.5	37.1 ± 8.8	6.8 ± 1.7	95.3

### 3.3 Clinical efficacy

**Figure 1:**
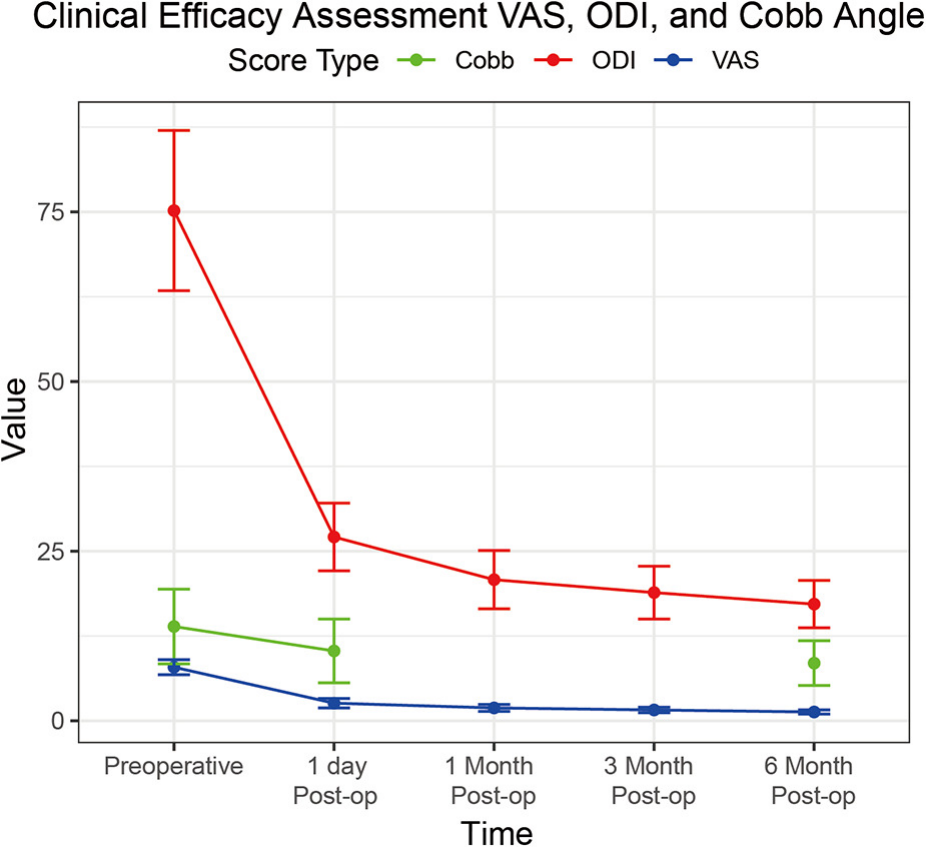
Line graph demonstrating the progression of VAS scores (blue line), ODI scores (red line), and cobb angle (green line) from preoperative baseline to six months post-operation.

Clinical efficacy results for VAS scores, ODI scores, and Cobb angles are in Table 3; Figure 1. The clinical efficacy analysis revealed significant temporal improvements across all measured parameters. VAS scores demonstrated a progressive reduction from 7.9 ± 1.1 at baseline to 1.3 ± 0.3 at 6 months postoperatively [*F* (4,504) = 386.2, *p* < 0.001, *η*² = 0.75], with an 83.5% overall improvement rate. ODI scores showed parallel trends, decreasing from 75.2 ± 11.8% to 17.2 ± 3.5% at final follow-up [*F* (4,504) = 412.8, *p* < 0.001, *η*² = 0.77], indicating sustained functional recovery. For Cobb angle, pairwise comparisons revealed a 5.4° correction from preoperative to 6 months [*t*(126) = 12.6, *p* < 0.001, *d* = 1.12], despite missing 1-/3-month imaging data. These findings collectively validate the intervention's capacity to achieve rapid pain relief, functional restoration, and anatomical recovery in a day-surgery context.

**Table 3:** Clinical efficacy assessment VAS ODI and cobb angle.

Index	Preoperative	1 day post-op	1 month post-op	3 months post-op	6 months post-op	Statistical values		
						*F*/*t*-value	*η*²/*d*	*p*-value
VAS Score	7.9 ± 1.1***	2.6 ± 0.7***	1.9 ± 0.5***	1.6 ± 0.4***	1.3 ± 0.3***	*F* (4, 504) = 386.2	*η*² = 0.75	*p* < 0.001
ODI Score (%)	75.2 ± 11.8***	27.1 ± 5.0***	20.8 ± 4.3***	18.9 ± 3.9***	17.2 ± 3.5***	*F* (4, 504) = 412.8	*η*² = 0.77	*p* < 0.001
Cobb Angle (°)	13.9 ± 5.5***	10.3 ± 4.7***	–	–	8.5 ± 3.3***	*t* (126) = 12.6	*d* = 1.12	*p* < 0.001

Data presented as mean ± SD; ***indicates *p* < 0.001 vs. preoperative (VAS and ODI analyzed by repeated measures ANOVA with Bonferroni correction; Cobb Angle analyzed by paired *t*-test). VAS/ODI: Significant time effects (*p* < 0.001) with large effect sizes (*η*² > 0.70).

### 3.4 Complications, hospital stay, and costs

The complication rate, length of hospital stay, and hospitalization costs are presented in Table 4. Three patients (2.4%) experienced bone cement leakage. The other complications (2.4%) included two cases of postoperative incision infection and one case of intraoperative transient hypotension. No patients experienced surgery- or fracture-related readmission within 30 days postoperatively. The mean length of hospital stay was 34 ± 4.3 h, and the mean total hospitalization cost was 37,100 ± 4,200 RMB.

**Table 4:** Complications, hospital stay, and costs.

Index	Cement leakage (*n*, %)	Other complications (*n*, %)	30-day readmission (*n*, %)	Hospital stay (hours)	Total cost (RMB)
Value	3 (2.4)	3 (2.4)	0 (0)	34 ± 4.3	37,100 ± 4,200

## 4 Discussion

OVCFs are frequently observed in elderly populations, with an increasing incidence correlated with global population aging. Investigations have demonstrated that these fractures not only induce substantial pain but can also result in protracted functional disabilities, thereby diminishing patients’ overall quality of life (16). Dual-energy x-ray absorptiometry serves as the primary diagnostic modality for osteoporosis, with a T-score of ≤−2.5 indicative of the condition. Elderly women exhibit heightened susceptibility to OVCFs; global projections suggest that approximately 25% of elderly women may sustain osteoporosis-related fractures by 2030 (12, 17). Consequently, identifying and implementing efficacious and safe therapeutic interventions is paramount for optimizing patient outcomes.

PKP has demonstrated favorable outcomes as a minimally invasive surgical technique for the management of OVCFs. Compared with conventional PKP, robot-assisted technology demonstrates significant advantages in both safety and efficacy. Owing to its enhanced operational precision, this approach substantially reduces perioperative tissue damage and lowers complication rates associated with neurovascular injuries, while achieving superior vertebral reduction and pain relief (18). Through preoperative three-dimensional modeling and trajectory simulation, robotic systems enable optimal puncture path planning that avoids critical anatomical structures, thereby minimizing puncture attempts and surgical trauma. This technological advancement not only decreases operative risks but also contributes to shorter procedure durations (19, 20). Additionally, robotic guidance systems significantly reduce intraoperative radiation exposure for both surgeons and patients, mitigating long-term radiation-associated hazards (21). The precision of robotic positioning further facilitates uniform cement distribution along the vertebral midline or fracture regions, providing enhanced pain control and structural support, thereby reducing leakage risks (18, 22). The integration of this advanced technology into orthopedic surgery has not only bolstered the safety and efficacy of surgical interventions but has also yielded substantial benefits for patients. Emerging evidence suggests potential advantages of robot-assisted percutaneous kyphoplasty (RA-PKP) over conventional fluoroscopy-guided PKP (FA-PKP) in clinical efficacy. Li et al. (23) demonstrated superior pain relief in RA-PKP patients, with VAS scores of 2.5 ± 0.5 vs. 2.6 ± 0.5 (1-day post-op) and 1.2 ± 0.2 vs. 1.5 ± 0.3 (3-month post-op) compared to FA-PKP. Our findings align with this trend, showing RA-PKP VAS scores of 2.6 ± 0.7 (1-day) and 1.9 ± 0.5 (3-month). While Liu et al. (24) reported comparable ODI improvements (RA-PKP: 24.09 ± 8.27 vs. FA-PKP: 24.77 ± 8.22), Lin et al. (25) observed slightly reduced Cobb angle correction with RA-PKP (9.19° ± 3.39° vs. 10.60° ± 2.69°), potentially influenced by patient selection heterogeneity. Our RA-PKP cohort achieved 8.5° ± 3.3° correction, warranting further investigation. Technical precision advantages are evident in RA-PKP's superior first-attempt success rate [95.3% in our study vs. 95.8% (26) and 63.2% for FA-PKP (26)]. Radiation exposure outcomes remain controversial: Li et al. (23) reported lower fluoroscopy counts (39.4 ± 8.2 vs. 70.2 ± 35.2), doses (222.2 ± 95.1 vs. 435.4 ± 119.4 mGy), and cement leakage rates (6.67% vs. 60.00%) for RA-PKP (*p* < 0.05). Conversely, Lin et al. (25) documented higher RA-PKP radiation (197.85 ± 31.21 vs. 123.00 ± 20.61 mGy), attributed to intraoperative continuous scanning for real-time registration (26, 27). Our protocol achieved balanced exposure (6.8 ± 1.7 fluoroscopy counts, 37.1 ± 8.8 mGy) through optimized robotic trajectory planning.

This study leverages the concept of day surgery in the treatment paradigm for OVCFs, introducing a novel perspective on osteoporosis management. The day-surgery model prioritizes discharging patients within 24 h of admission (extendable to 48 h contingent upon national guidelines in China). Through the optimization of preoperative assessment and postoperative care, this model can substantially reduce hospital stays, improve bed utilization rates, and alleviate the demands on hospital resources (28). Concurrently, systematic reviews have indicated that emergency day surgery can mitigate the burden of emergency surgical procedures while capitalizing on the advantages of day surgery, thereby constituting an innovative approach to emergency perioperative management (29). The conclusions drawn from these investigations lend theoretical credence to the present study, suggesting that the successful implementation of emergency day surgery models in other disease contexts may be transferable to the treatment of OVCFs, potentially extending to emergency OVCF patients in the future. The current study proactively incorporates the FLS, a systematic framework for osteoporosis management designed to curtail the risk of refractures in individuals with fragility fractures. A key strength of the Kunshan model lies in its multidisciplinary collaborative approach, integrating expertise from the fields of orthopedics, endocrinology, rehabilitation, and nutrition. The synergistic combination of FLS and the day-surgery model facilitates the integration of acute care with sustained osteoporosis management for patients, fostering rapid recovery and enduring benefits. It is noteworthy that prior investigations have seldom addressed the synergistic effects of day surgery, robot-assisted technology, and FLS. The findings of this study furnish preliminary evidence supporting the combined application of these three modalities. This paradigm not only empowers patients to recuperate expeditiously within their home environment but also mitigates the risk of nosocomial infections and contributes to enhanced patient satisfaction. Our observations indicate that patients undergoing day surgery in conjunction with robot-assisted technology for OVCFs exhibit favorable outcomes during postoperative follow-up, with no reported surgery-related readmissions within 30 days, thereby substantiating the effectiveness of the day-surgery model in clinical practice. Furthermore, all surgical procedures in this study were conducted under local anesthesia, thereby obviating the risks potentially associated with general anesthesia. Unilateral puncture reduces operation time and improves patients’ tolerance to body position, which is especially more prominent for elderly OVCF patients with multiple underlying diseases.

The findings of this study substantiate that RA-PKP under local anesthesia in a day-surgery setting confers notable benefits in the management of OVCFs. Postoperative assessments revealed a marked reduction in VAS scores, coupled with a 77.1% improvement rate in ODI, underscoring the capacity of this technique to effectively alleviate pain and enhance functional capabilities in patients. Notably, the cement leakage rate within the robot-assisted cohort was substantially lower than rates previously documented, primarily attributable to the precise positioning and puncture path planning facilitated by the robotic system. This allowed surgeons to exercise greater control over the placement and dosage of cement injection, thereby mitigating the risk of leakage and consequent iatrogenic injury. The initial puncture success rate in this study reached 95.3%, unequivocally demonstrating the accuracy afforded by robot-assisted technology. Crucially, robot-assisted technology significantly enhanced the precision of balloon dilation, achieving bone cement distribution patterns akin to those obtained with a pouch, thus presenting a viable alternative for selected cases of vertebral burst fractures. Unilateral puncture further contributed to reduced material consumption, subsequently lowering medical expenditures. Although the hospital stays of most patients surpassed 24 h, it was observed that all patients were discharged within 48 h, aligning with the day-surgery model as adapted to the healthcare context in China. Looking ahead, ongoing efforts to refine day-surgery protocols, bolster postoperative pain management strategies, and enhance patient education initiatives hold promise for further optimizing the efficiency of day-surgery practices.

### 4.1 Limitations

Several limitations warrant consideration. The single-center, retrospective design, modest sample size, and lack of randomized controls may affect generalizability. Future studies should use larger, multi-center prospective designs. The focus on short-term outcomes precluded evaluation of long-term results like refracture rates and quality of life, which future research should address. Furthermore, costs and technical complexities of robotic systems may limit widespread implementation. Although this study lacks direct comparisons with FA-PKP, emerging evidence suggests that RA-PKP may demonstrate potential advantages in pain relief, functional recovery, and Cobb angle correction. Future research should compare RA-PKP and FA-PKP regarding long-term clinical efficacy, complication incidence, and cost-effectiveness to inform evidence-based clinical decision-making.

## 5 Conclusion

With strict adherence to selection criteria, thorough preoperative preparation, and expert clinical assessment, robot-assisted PKP under local anesthesia within a day-surgery setting is a safe and effective treatment option for carefully selected patients with OVCFs.

## Data Availability

The original contributions presented in the study are included in the article/Supplementary Material, further inquiries can be directed to the corresponding author.
